# Eosinophil Levels, Neutrophil-Lymphocyte Ratio, and Platelet-Lymphocyte Ratio in the Cytokine Storm Period of Patients with COVID-19

**DOI:** 10.1155/2022/7450739

**Published:** 2022-08-03

**Authors:** Ibrahim Koc, Sevda Unalli Ozmen

**Affiliations:** ^1^Bursa City Hospital, Pulmonary Medicine Clinic, Bursa, Turkey; ^2^Bursa City Hospital Medical Biochemistry, Bursa, Turkey

## Abstract

**Background:**

In the early stages of the COVID-19 pandemic, elevated inflammatory cytokine levels, particularly interleukin-6 (IL-6), were detected in patients with cytokine storm (CS).

**Aims:**

This study aimed to investigate levels, diagnostic usefulness, and optimal cutoff values of monocyte, eosinophil, neutrophil-lymphocyte ratio (NLR), and platelet-lymphocyte ratio (PLR) in CS of patients with COVID-19 and also to identify risk factors for mortality.

**Methods:**

Seventy-six patients with COVID-19 who developed CS and randomly chosen 150 COVID-19 patients who had no CS during their stay in the hospital were included in the study.

**Results:**

Lymphocytes and eosinophil levels remained lower in the CS group. Patients with low lymphocyte levels had a higher risk for mortality (OR: 1.92). Neutrophil, D-dimer, ferritin, IL-6, NLR, and PLR were higher in the CS group. High levels of neutrophil, ferritin, D-dimer, and NLR and a history of coronary artery disease (CAD) and diabetes mellitus (DM) were identified as independent risk factors for mortality.

**Conclusion:**

In the light of the obtained results, COVID-19 patients with a decrease in lymphocyte levels and an increase in NLR and D-dimer levels and a history of CAD and DM have a higher risk of cytokine storm and mortality.

## 1. Introduction

A new disease was announced at the end of 2019, coronavirus disease-2019 (COVID-19), caused by the severe acute respiratory syndrome coronavirus-2 (SARS-CoV-2). COVID-19 has been accused of the death of millions since its identification. In the early stages of the pandemic, elevated inflammatory cytokines, particularly interleukin-6 (IL-6), were detected in patients with a bad prognosis [1]. High levels of IL-6 were also reported in asthmatic subjects [2]. The COVID-19 cytokine storm (CS) is a life-threatening condition associated with increased circulating cytokine levels and immune cell hyperactivation. Previous research studies supported immunosuppressive therapies such as cytokine blockade, which is effective in treating COVID-19 CS [3]. Tocilizumab is a medicine that is mainly used to treat rheumatoid arthritis and systemic juvenile idiopathic arthritis [4]. It has been beneficial in critically ill patients, proof of using immunosuppressive therapies and intravenous immunoglobulin in some patients with COVID-19 [5, 6]. There are no specific criteria, cutoff levels, or definitions for diagnosing the cytokine storm. Neutrophil-lymphocyte ratio (NLR), eosinophil count, and platelet-lymphocyte ratio (PLR) are considered novel predictors of inflammation. Previous studies reported high levels of neutrophil-lymphocyte ratio in native septic arthritis [7], lymph node metastasis of esophageal squamous cell carcinoma [8], and COVID-19 infection [9]. The red blood cell distribution width (RDW) has been investigated as a diagnostic and prognostic biomarker [10]. On the other hand, elevated levels of platelet-lymphocyte ratio were reported in acute limb ischemia [11], malignant diseases [12], and COVID-19 infection [13].

This study aimed to determine whether eosinophil count, platelet distribution width (PDW), RDW, NLR, PLR, and monocyte-lymphocyte ratio (MLR) could diagnose cytokine storms in laboratory-confirmed COVID-19 patients, analyze optimal cutoff values, and estimate the risk for mortality.

## 2. Materials and Methods

### 2.1. Study Design and Data Collection

The files of 5126 patients admitted to a tertiary hospital in Turkey between March 2020 and August 2020 with complaints compatible with COVID-19 were investigated. The patients' demographic, clinical, and laboratory (complete blood count, biochemistry) data were retrospectively obtained from the hospital data management system. The patients with one of the criteria comprising oxygen saturation below 93% in room air, C-reactive protein (CRP) value above 50 mg/L, D-dimer value above 1 ug FEU/ml, ferritin level above 500 ng/mL, lymphocyte value below 500 × 10^3^/*μ*L, and severe involvement on computed tomography were hospitalized. Patients with and without CS were compared by examining the hemogram and biochemical parameters obtained at the time of CS diagnosis and the control group at the first admission to the hospital. CRP and D-dimer were analyzed using the particle-enhanced immunoturbidometric essay. Ferritin levels were assayed using the sandwich principle. Leukocytes were measured using fluorescent flow cytometry and erythrocytes and platelets using the impedance method. Ratios of neutrophil-lymphocyte, platelet-lymphocyte, and monocyte-lymphocyte were calculated by dividing neutrophil, platelet, and monocyte levels by lymphocyte count.

### 2.2. Inclusion and Exclusion Criteria

Seventy-six patients with a progressive increase in C-reactive protein (CRP), D-dimer, ferritin, and IL-6 levels, no growth on blood, urine, and sputum cultures, and considered to have CS by 3 physicians (pulmonary medicine specialist, infectious diseases specialist, and rheumatology or hematology specialist) and 150 patients who did not have CS during the hospital stay were accepted for inclusion in the study. The control group was randomly selected from patients hospitalized in the same clinics simultaneously. Pregnant women and patients with immunosuppressives, rheumatic diseases, malignancies, and insufficient data were excluded.

### 2.3. Ethics Committee Approval

After obtaining scientific research approval for the study from the Ministry of Health General Directorate of Health Services, the ethics committee approval was obtained from the Clinical Research Ethics Committee of a tertiary city hospital in Turkey (ethics committee approval no. 2021-12/1).

### 2.4. Statistical Analysis

The statistical analyses were performed using SPSS 25.0 software. The normality of the sample data was evaluated with the Kolmogorov–Smirnov test, and the continuous variables were defined by the mean ± standard deviation, median (minimum-maximum), and categorical variables were expressed as frequency and percent. The independent groups were compared using Student's *t*-test for parametric assumptions and the Mann–Whitney *U* test for nonparametric assumptions. The ROC analysis was performed to predict optimal cutoff values for CS, and a *p* value less than 0.05 was set as the statistical significance level. The multivariate binary logistic regression was used to identify independent predictors associated with in-hospital mortality.

## 3. Results

The mean age was found as 62.7 ± 1.3 in the CS group and 49 ± 1.6 in the non-CS group (Table 1). The mean age was significantly higher in the CS group (*p* < 0.001). Although the gender distribution in the CS group was 20 (26.3%) women and 56 (73.6%) men, it was 70 (46.6%) women and 80 (53.3%) men in the non-CS group.

Diabetes mellitus (DM), coronary artery disease (CAD), intensive care unit (ICU) stay, and death rates were higher in the CS group (all *p* < 0.001), whereas the percentage saturation of oxygen in blood (SpO_2_) at the time of admission to the hospital and clinic stay was higher in the non-CS group (*p* < 0.001). Lymphocyte, monocyte, and eosinophil levels remained significantly lower in the CS group (all *p* < 0.001). In contrast, neutrophil levels, platelets, RDW-SD, D-dimer, ferritin, IL-6, CRP, NLR, PLR, and MLR were higher in the same group (all *p* < 0.001) (Table 1).

**Figure 1:**
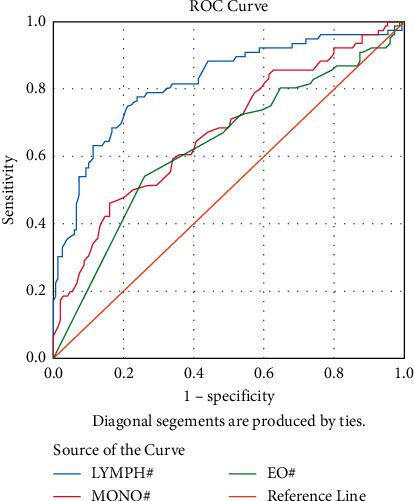
ROC curves comparing the prediction of COVID-19 with cytokine storm variables for lymphocyte (LYMPH), monocyte (MONO), and eosinophil (EO).

**Figure 2:**
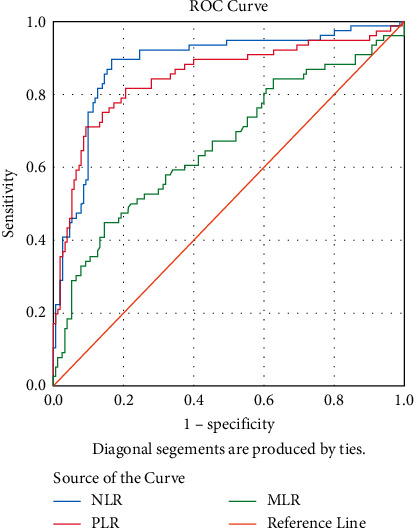
ROC curves comparing the prediction of COVID-19 with cytokine storm variables for neutrophil-lymphocyte ratio (NLR), platelet-lymphocyte ratio (PLR), and monocyte-lymphocyte ratio (MLR).

The ROC analysis with ROC curves was performed for optimal cutoff values to predict CS.  Figure 1 shows ROC curves for lymphocyte (LYMPH), monocyte (MONO), and eosinophil (EO).  Figure 2 shows ROC curves for neutrophil-lymphocyte ratio (NLR), platelet-lymphocyte ratio (PLR), and monocyte-lymphocyte ratio (MLR). The areas under the curve (AUC) of lymphocyte, monocyte, eosinophil, NLR, PLR, and MLR were found as 0.81, 0.67, 0.63, 0.88, 0.84, and 0.66, respectively, and all *p* < 0.001 (Table 2).

Binary logistic regression analysis was performed to predict mortality risk, and odds ratios for statistically significant results were calculated (Table 3). Variables independently associated with an increased mortality risk were higher levels of neutrophil (OR: 1.37 (95% CI: 1.24–1.52; *P*=0.001)), NLR (OR: 1.25 (95% CI: 1.11–1.42; *P*=0.001)), IL-6 (OR: 1.001 (95% CI: 1–1.001; *P*=0.032)), ferritin (OR: 1.001 (95% CI: 1–1.002; *p*=0.034)), and D-dimer (OR: 1.36 (95% CI: 1.016–1.83; *p*=0.039)) and lower levels of lymphocyte (OR: 1.9 (95% CI: 1.9–3.3; *P*=0.024)). The patients with higher PLR (OR: 0.99 (95% CI: 0.99–0.999;*P*=0.025)) and lower eosinophil levels (OR: 0.01 (95% CI: 0–0.4; *P*=0.015)) also had a decreased mortality risk. Patients with a history of CAD had an odds ratio of 6.9, DM 4.4, and increased age of 1.04 and had an adjusted multivariable for mortality risk. The multivariable model had an area under the receiver operating characteristic curve estimate of 0.86 (95% CI: 0.83–0.92).

## 4. Discussion

The present study found that neutrophil, lymphocyte, monocyte, eosinophil, NLR, PLR, and MLR of patients with CS were significantly different from those without CS. We hypothesized that these differences might help in identifying COVID-19 patients with CS.

Lymphocytes are essential cells in the maintenance of immune system function. Following viral infections, changes in total lymphocyte numbers vary with different virus types. According to studies, lower lymphocytes may help distinguish asymptomatic COVID-19 from the moderate disease [14]. Else, lower lymphocyte counts were identified in COVID-19 patients who required intubation and patients who died [15]. The CS group had lower lymphocyte levels associated with increased mortality risk in the present study. This finding was compatible with the data in the literature regarding COVID-19 and added to our knowledge about COVID-19 patients with CS.

Neutrophils are innate immune cells that have a short lifespan after leaving the bone marrow and play a crucial role in the first line of cell-mediated defense against microbes [16]. Higher neutrophil levels were reported in severe COVID-19 patients compared to mild and moderate disease [17]. In the present study, neutrophil levels were higher in the CS group and were associated with increased mortality risk (OR: 1.37).

Recent studies highlighted the predominant role of monocytes in developing immunopathology of COVID-19 [18]. Zhang et al. reported morphological and inflammation-based phenotypic changes in peripheral blood monocytes of COVID-19 patients, which correlated with the patient's outcome [19]. In another study, in patients with severe COVID-19, a reduced rate of activated monocytes was reported [20]. In the present study, low monocyte levels were found in the CS group. In the light of the current research and the studies mentioned above, monocytes might play an essential role in COVID-19 disease.

Eosinophils are cells that have various functions such as immunoregulation and antiviral activity. Yet, their role in COVID-19 during the CS is not well known. Zhang et al. have previously reported eosinopenia in patients with acute respiratory deterioration during infection with SARS-CoV-2 [21]. In another study, eosinopenia was reported more frequently among moderate and severe COVID-19 patients [22]. Consistent with the previous studies, in the present study, eosinophil values were lower in the CS group. But interestingly, in the present study, the lower eosinophil levels were associated with a decreased mortality risk (OR: 0.01). We do not know the exact reason, but it might be because COVID-19 is a heterogeneous disease and has different clinical and laboratory presentations.

Research workers have investigated some ratios as diagnostic and prognostic markers of inflammatory diseases. Some of these include neutrophil-lymphocyte and platelet-lymphocyte ratios. In a study, Seyit et al. reported a high NLR in COVID-19 patients compared to controls [13]. In another study, NLR values were significant in the diagnosis of COVID-19 [9]. In a study from Wuhan/China, Yang et al. reported that elevated NLR was associated with illness severity [23]. In the present study, the CS group had high levels of NLR, which was an independent risk factor for mortality (OR: 1.25). Higher levels were associated with high sensitivity and specificity in predicting CS (AUC: 0.88, sensitivity 85%, and specificity 84%).

The monocyte-lymphocyte ratio is an essential parameter that has recently been studied.. High levels were reported in COVID-19 patients [24]. Another study reported higher levels of MLR in COVID-19 patients admitted to the intensive care unit [25]. In accordance with previous results in this study, MLR was higher in the CS group, which could be used as a predictive diagnostic tool for CS and determining patients who require ICU support.

PLR is another ratio that has recently been the subject of research. The present study demonstrated that high PLR in the CS group was associated with a slightly decreased mortality risk (OR: 0.99). Like NLR, high PLR was associated with high sensitivity and specificity in predicting CS (AUC: 0.84 sensitivity 80%, and specificity 79%). In a study, Yang et al. reported high PLR in severe COVID-19 compared to nonsevere COVID-19 patients [23]. A previous study reported more elevated PLR in patients in the intensive care unit [25]. The results of our study are consistent with the studies mentioned above and suggest that PLR values may be a guide in the diagnosis of CS.

The red blood cell distribution width measures the range of red blood cell volume variation reported as a part of the standard complete blood count. High RDW levels were reported as prognostic predictors for patients with severe COVID-19 [26]. Our study has demonstrated the higher levels of RDW-SD in the CS group. Similar to this study, Henry et al. discovered a progressive increase in RDW with advancing COVID-19 severity [27]. In the light of the present research and studies mentioned above, higher levels of RDW-SD might indicate a CS in patients with COVID-19.

In critically ill patients, COVID-19 is associated with an inflammatory cytokine storm in which the inflammatory response may change the iron homeostasis. Zhou et al. reported higher serum ferritin levels in patients diagnosed with severe COVID-19 than in other groups [28]. According to their results in our study, serum ferritin levels were higher in the CS group (*p* < 0.001). In previous studies, high levels of PDW have been related to COVID-19 mortality [29]. In the present study, no difference was observed between groups. Although the results of our study suggest that RDW values will not aid in the diagnose CS, more studies are needed to elucidate the issue.

Our study has some limitations, the most significant of which are its retrospective nature and single-center design. Although the blood values of the control group belong to the pretreatment period, the blood parameters, including lymphocyte, eosinophil, and neutrophil levels, of the CS group may have been affected by treatments such as corticosteroids. The age and gender differences between the groups were the study's limitations and may have affected the results.

## 5. Conclusions

Based on the obtained results, a decrease in lymphocyte, monocyte, and eosinophil levels and an increase in NLR, PLR, and MLR should raise suspicion for CS, especially in clinics where IL-6 is not available. Patients with higher NLR, D-dimer, a history of diabetes mellitus, and coronary artery disease should be followed closely as their mortality risk seems to be higher.

## Figures and Tables

**Table 1:** Demographic, clinical data, and laboratory findings of COVID-19 patients with and without cytokine storm.

Parameters	CS (*n* = 76)	No CS (*n* = 150)	*P* value
Age (years)	62.7 ± 1.3	49 ± 1.6	0.001^*∗*^
Number of females	20 (26.3%)	70 (48.7%)	0.001^*∗*^
COPD, *n* (%)	2 (2.6%)	1 (0.7%)	0.22
DM, *n* (%)	24 (31.6)	14 (9.3)	0.001^*∗*^
CAD, *n* (%)	25 (32.9)	8 (5.3)	0.001^*∗*^
SpO_2_ (%)	92 (74–98)	96 (75–98)	0.001^*∗*^
Clinic stay (day)	4 (0–33)	9 (0–40)	0.001^*∗*^
ICU stay (day)	15 (0–65)	3.2 (0–61)	0.001^*∗*^
Death, *n* (%)	48 (63.2%)	17 (11.3%)	0.001^*∗*^
Neutrophil, 10^3^/L	8.1 (1.7–38)	4.06 (0.37–22)	0.001^*∗*^
Lymphocyte, 10^3^/L	0.68 (0.14–9.5)	1.43 (0.39–9.8)	0.001^*∗*^
Monocyte. 10^3^/L	0.37 (0.01–1.231)	0.51 (0.14–1.44)	0.001^*∗*^
Eosinophil, 10^3^/L	0.00 (0–0.55)	0.02 (0–1.45)	0.001^*∗*^
PLT, 10^3^/L	275 (33–824)	222 (96–623)	0.001^*∗*^
RDW-SD, fL	42 (33–76)	39 (30–66)	0.001^*∗*^
PDW, fL	11.9 ± 1.9	12.1 ± 2.04	0.6
D-Dimer, ug FEU/ml	1.8 (0.32–19)	0.42 (0.15–16)	0.001^*∗*^
Ferritin, ng/mL	968 (140–2898)	265 (8.2–1941)	0.001^*∗*^
IL-6, pg/mL	663 (38–24512)	48 (8–691)	0.001^*∗*^
CRP, mg/L	116 (0.9–372)	18 (0.3–434)	0.001^*∗*^
NLR	11 (0.53–72)	2.5 (0.44–25)	0.001^*∗*^
PLR	408 (29–3021)	151 (27–714)	0.001^*∗*^
MLR	0.52 (0.03–2.09)	0.36 (0.1–1.6)	0.001^*∗*^

COPD, chronic obstructive pulmonary disease; DM, diabetes mellitus; CAD, coronary artery disease; SpO_2_, the percent saturation of oxygen in blood; ICU, intensive care unit; PLT, platelets; IL-6, interleukin-6; NLR, neutrophil-lymphocyte ratio; PLR, platelet-lymphocyte ratio; MLR, monocyte-lymphocyte ratio. ^*∗*^*P* < 0.05, statistically significant.

**Table 2:** ROC analysis of COVID-19 patients with and without cytokine storm.

Variables	AUC (95% CI)	Cutoff	Sensitivity (%)	Specificity (%)	*P* value
Lymphocyte	0.81 (75−88)	≤0.99	76	76	0.001^*∗*^
Monocyte	0.67 (0.59−0.75)	≤0.47	61	60	0.001^*∗*^
Eosinophil	0.63 (0.55−0.71)	≤0.085	57	51	0.001^*∗*^
NLR	0.88 (0.83−0.93)	≥5.9	85	84	0.001^*∗*^
PLR	0.84 (0.78−0.9)	≥216	80	79	0.001^*∗*^
MLR	0.66 (0.58−0.74)	≥0.4	60	59	0.001^*∗*^

AUC, area under the ROC curve; NLR, neutrophil-lymphocyte ratio; PLR, platelet-lymphocyte ratio; MLR, monocyte-lymphocyte ratio. ^*∗*^*P* < 0.05, statistically significant.

**Table 3:** Binary logistic regression model demonstrating odds ratios of statistically significant variables predicting mortality.

Variables	OR	*P* value
Neutrophil	1.37 (1.24−1.52)	0.001^*∗*^
Lymphocyte	1.922 (1.09−3.38)	0.024^*∗*^
Eosinophil	0.010 (0−0.4)	0.015^*∗*^
Platelets	1.010 (1.001−1.01)	0.030^*∗*^
NLR	1.258 (1.15−1.42)	0.001^*∗*^
PLR	0.995 (0.99−0.99)	0.025^*∗*^
IL-6	1.001 (1-1.001)	0.032^*∗*^
Ferritin	1.001 (1.05−1.06)	0.034^*∗*^
D-Dimer	1.365 (1.016−1.8)	0.039^*∗*^
CAD	6.795 (1.77−25)	0.005^*∗*^
DM	4.4 (2.1−9.3)	0.001^*∗*^
Age	1.040 (1.01−1.06)	0.005^*∗*^

Data in parentheses are 95% confidence intervals. OR, odds ratio; NLR, neutrophil-lymphocyte ratio; PLR, platelet-lymphocyte ratio; CAD, coronary artery disease; DM, diabetes mellitus. ^*∗*^*P* < 0.05, statistically significant.

## Data Availability

The data used to support this study are not available due to ethical restrictions.

## References

[1] Chen L. Y. C., Hoiland R. L., Stukas S., Wellington C. L., Sekhon M. S. (2020). Confronting the controversy: interleukin-6 and the COVID-19 cytokine storm syndrome. *European Respiratory Journal*.

[2] Ceylan E., Bulut S., Yilmaz M. (2019). The levels of serum biomarkers in stable Asthma patients with comorbidities. *Iranian Journal of Allergy, Asthma and Immunology*.

[3] Kesmez Can F., Ozkurt Z., Ozturk N., Sezen S. (2021). Effect of IL-6, IL-8/CXCL8, IP-10/CXCL 10 levels on the severity in COVID 19 infection. *International Journal of Clinical Practice*.

[4] Poddighe D., Romano M., Gattinara M., Gerloni V. (2019). Biologics for the treatment of juvenile idiopathic arthritis. *Current Medicinal Chemistry*.

[5] Gordon A. C., Angus D. C., Derde L. P. G. (2021). Interleukin-6 receptor antagonists in critically ill patients with covid-19. *The New England Journal of Medicine*.

[6] Bektas S., Ayaz C., Yüksel M. E., Izdes S. Evaluation of the efficacy of intravenous immunoglobulin therapy in coronavirus-19 patients followed in the intensive care unit.

[7] Varady N. H., Schwab P. E., Kheir M. M., Dilley J. E., Bedair H., Chen A. F. (2022). Synovial fluid and serum neutrophil-to-lymphocyte ratio: novel biomarkers for the diagnosis and prognosis of native septic arthritis in adults. *Journal of Bone and Joint Surgery*.

[8] Ohsawa M., Hamai Y., Emi M. (2022). Blood biomarkers as predictors of pathological lymph node metastasis in clinical stage T1N0 esophageal squamous cell carcinoma. *Diseases of the Esophagus*.

[9] Nalbant A., Kaya T., Varim C., Yaylaci S., Tamer A., Cinemre H. (2020). Can the neutrophil/lymphocyte ratio (NLR) have a role in the diagnosis of coronavirus 2019 disease (COVID-19)?. *Revista da Associação Médica Brasileira*.

[10] Gisondi P., Geat D., Lippi G., Montagnana M., Girolomoni G. (2021). Increased red blood cell distribution width in patients with plaque psoriasis. *Journal of Medical Biochemistry*.

[11] Arbănași E. M., Mureșan A. V., Coșarcă C. M. (2022). Neutrophil-to-Lymphocyte ratio and platelet-to-lymphocyte ratio impact on predicting outcomes in patients with acute limb ischemia. *Life*.

[12] Atak Tel B. M., Kahveci G., Bilgin S., Kurtkulagi O., Kosekli M. A. (2021). Platelet to lymphocyte ratio in differentiation of benign and malignant thyroid nodules. *Experimental Biomedical Research*.

[13] Seyit M., Avci E., Nar R. (2021). Neutrophil to lymphocyte ratio, lymphocyte to monocyte ratio and platelet to lymphocyte ratio to predict the severity of COVID-19. *The American Journal of Emergency Medicine*.

[14] Gu X., Sha L., Zhang S., Shen D., Zhao W., Yi Y. (2021). Neutrophils and lymphocytes can help distinguish asymptomatic COVID-19 from moderate COVID-19. *Frontiers in Cellular and Infection Microbiology*.

[15] Illg Z., Muller G., Mueller M., Nippert J., Allen B. (2021). Analysis of absolute lymphocyte count in patients with COVID-19. *The American Journal of Emergency Medicine*.

[16] Lamichhane P. P., Samarasinghe A. E. (2019). The role of innate leukocytes during influenza virus infection. *Journal of Immunology Research*.

[17] Eijmael M., Janssens N., le Cessie S., van Dooren Y., Koster T., Karim F. (2021). Coronavirus disease 2019 and peripheral blood eosinophil counts: a retrospective study. *Infection*.

[18] Gomez-Rial J., Curras-Tuala M. J., Rivero-Calle I. (2020). Increased serum levels of sCD14 and sCD163 indicate a preponderant role for monocytes in COVID-19 immunopathology. *Frontiers in Immunology*.

[19] Zhang D., Guo R., Lei L. COVID-19 Infection induces readily detectable morphological and inflammation-related phenotypic changes in peripheral blood monocytes. *Journal of Leukocyte Biology*.

[20] Kos I., Balensiefer B., Lesan V. (2021). Increased B-cell activity with consumption of activated monocytes in severe COVID-19 patients. *European Journal of Immunology*.

[21] Zhang J. J., Dong X., Cao Y. Y. (2020). Clinical characteristics of 140 patients infected with SARS-CoV-2 in Wuhan, China. *Allergy*.

[22] Priyanka P., Krishnamurthy V., Kumar T. A. (2022). A study to evaluate the role of eosinophil count as a prognostic marker for assessing the outcome in patients with COVID 19 infection. *Journal of the Association of Physicians of India*.

[23] Yang A. P., Liu J. P., Tao W. Q., Li H. M. (2020). The diagnostic and predictive role of NLR, d-NLR and PLR in COVID-19 patients. *International Immunopharmacology*.

[24] Bastug A., Bodur H., Erdogan S. (2020). Clinical and laboratory features of COVID-19: predictors of severe prognosis. *International Immunopharmacology*.

[25] Şener G., Bayrak T., Coşkun C., Bayrak A. (2022). Neutrophil lymphocyte ratio, monocyte lymphocyte ratio, platelet lymphocyte ratio in covid-19 patients. *Clinical Laboratory*.

[26] Wang F., Nie J., Wang H. (2020). Characteristics of peripheral lymphocyte subset alteration in COVID-19 pneumonia. *The Journal of Infectious Diseases*.

[27] Henry B. M., Benoit J. L., Benoit S. (2020). Red blood cell distribution width (RDW) predicts COVID-19 severity: a prospective, observational study from the cincinnati SARS-CoV-2 emergency department cohort. *Diagnostics*.

[28] Zhou C., Chen Y., Ji Y., He X., Xue D. (2020). Increased serum levels of hepcidin and ferritin are associated with severity of COVID-19. *Medical Science Monitor*.

[29] Lorente L., Martin M. M., Argueso M. (2021). Association between red blood cell distribution width and mortality of COVID-19 patients. *Anaesthesia Critical Care & Pain Medicine*.

